# P-1816. Malaria and West-Nile Virus Co-infection amongst Febrile Patients attending a Tertiary Hospital in Abuja, Nigeria

**DOI:** 10.1093/ofid/ofaf695.1985

**Published:** 2026-01-11

**Authors:** Kehinde Oluwasegun Aina

**Affiliations:** University of Ilorin, Nigeria, Braunschweig, Niedersachsen, Germany

## Abstract

**Background:**

West Nile virus (WNV) has ubiquitous distribution in Africa. Over the years, the geographical range of WNV activity has increased and the virus has become established even in non-endemic areas where it has not been previously detected.

**Figure:**
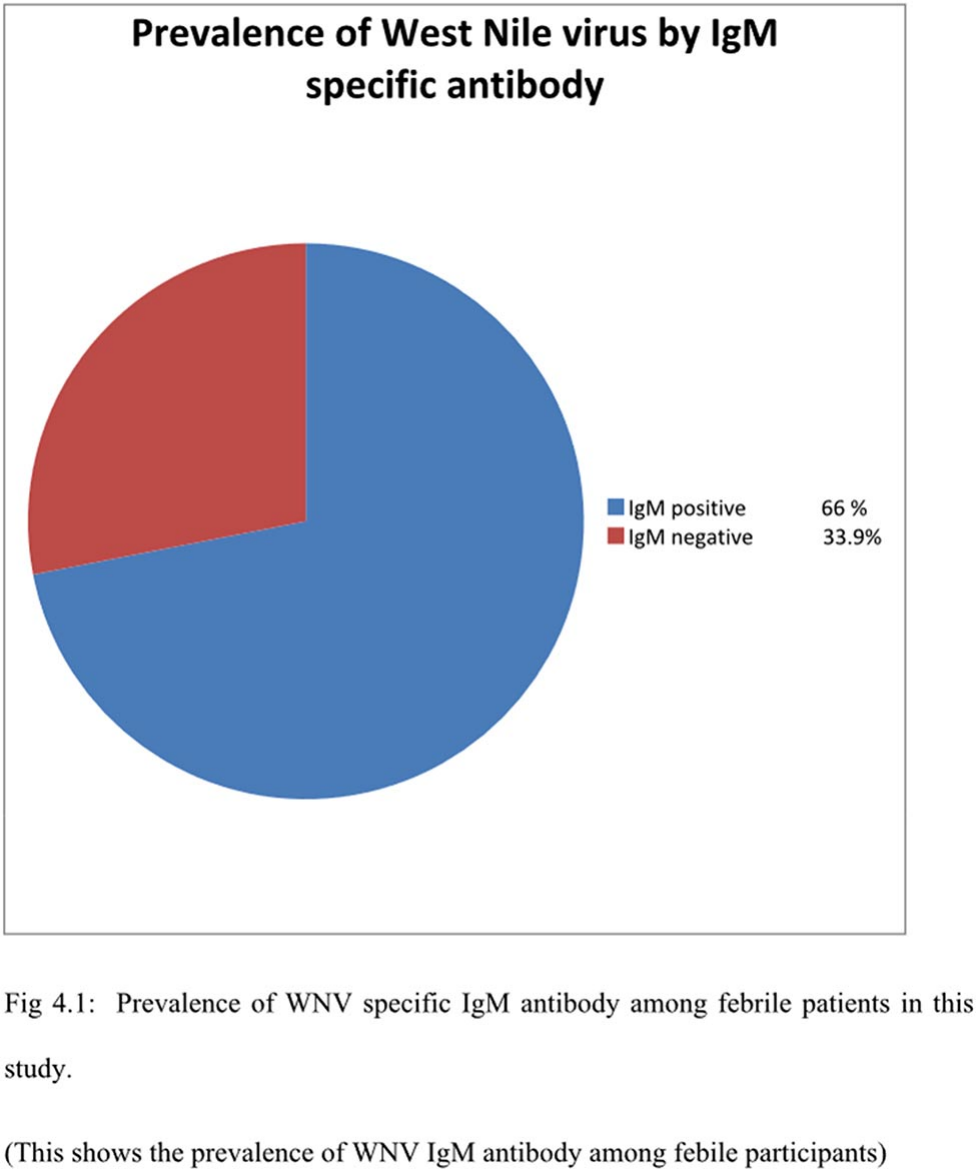

**Figure:**
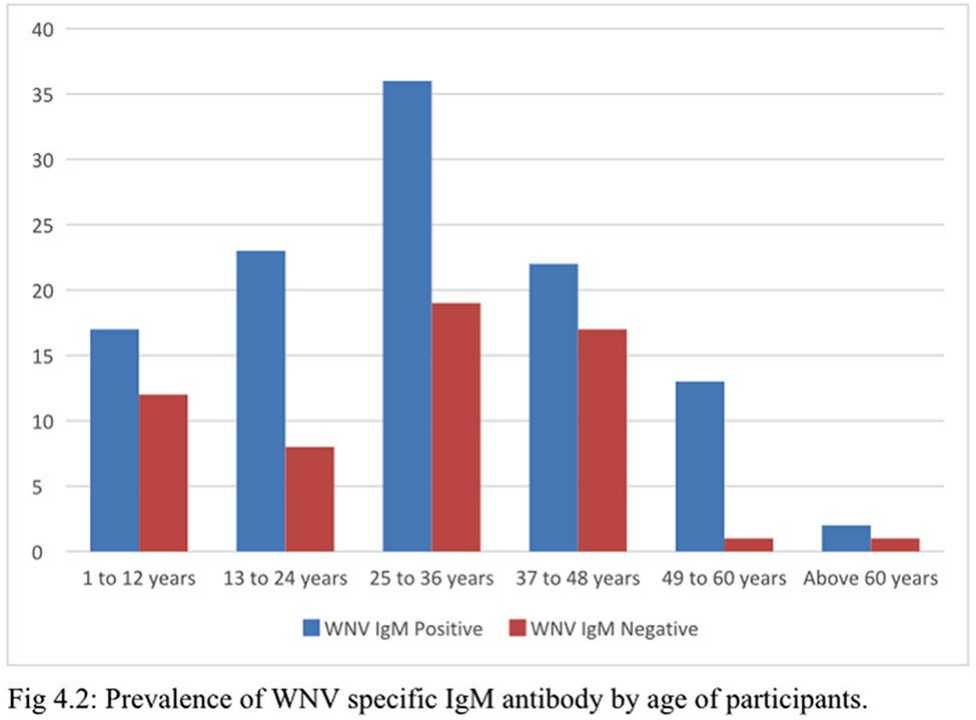

**Methods:**

This serological-survey investigated the prevalence of anti-WNV IgM among patients with febrile illnesses at Gwagwalada metropolis, Abuja. Between the period of May and August 2016, a total of 171 patients attending the University of Abuja Teaching Hospital were recruited for the study. Serum samples were immediately harvested, stored and analyzed using the indirect ELISA for anti-WNV IgM antibodies using kits endorsed by the World Health Organization. Socio-demographic variables and clinical data was gotten using a self-administered interviewer-based questionnaires.

**Figure:**
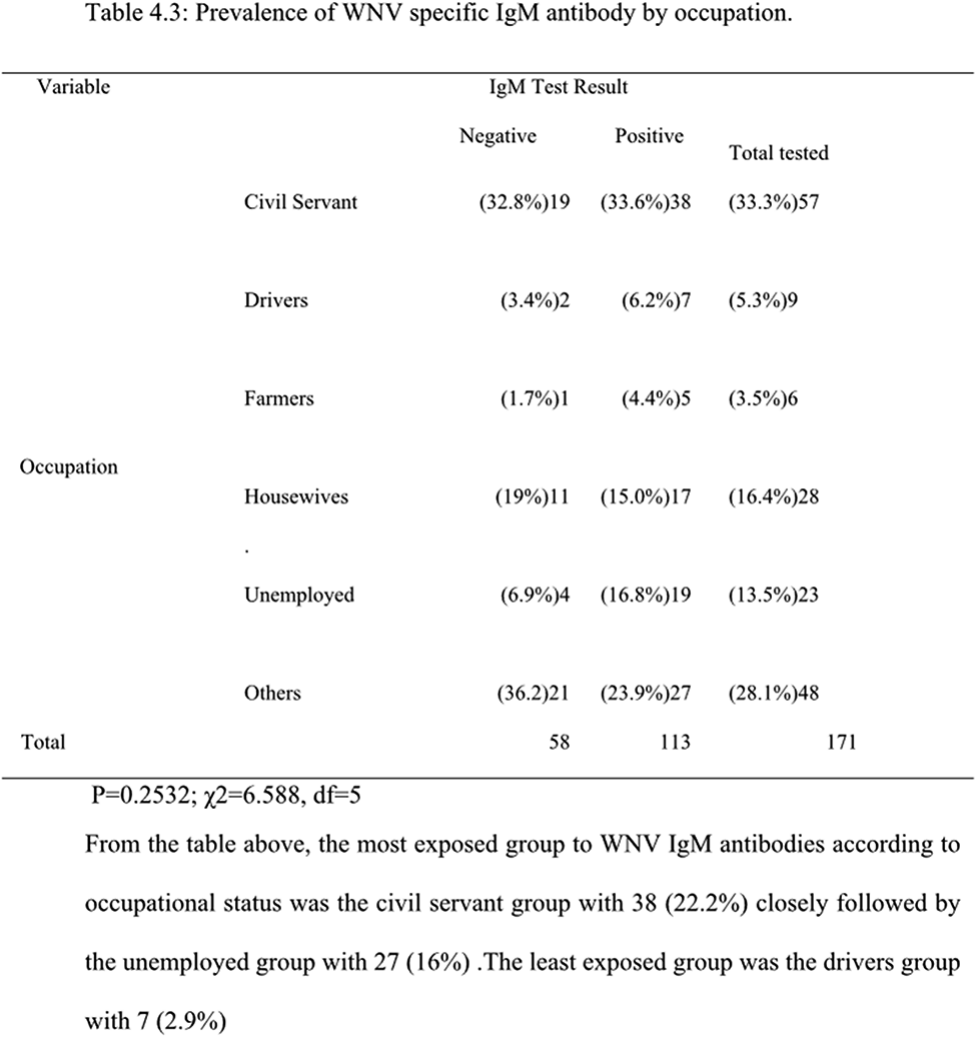

**Figure:**
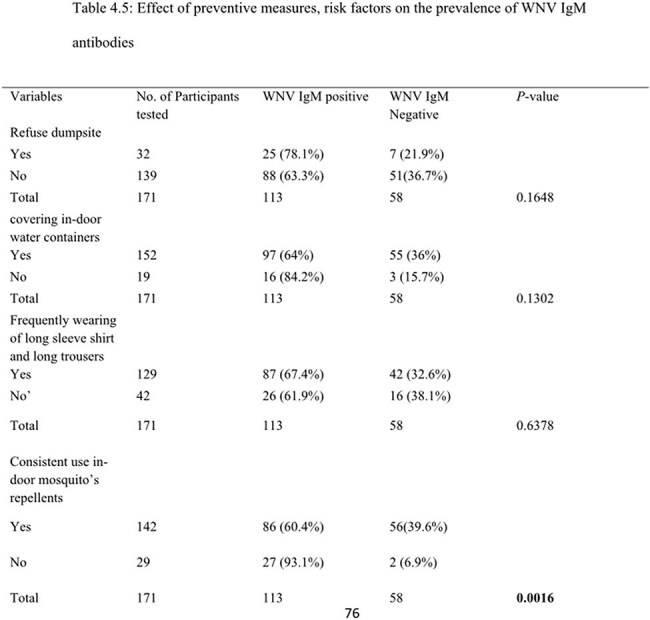

**Results:**

Out of the 171 febrile participants, the overall prevalence of WNV IgM antibodies was 66.1%. With regards to participants preventive measures against WNV and associated risk factors, significant association was observed between WNV IgM seropositivity and the use of mosquito repellents (p =0.016).

**Conclusion:**

Out of the 171 febrile participants, the overall prevalence of WNV IgM antibodies was 66.1%. With regards to participants preventive measures against WNV and associated risk factors, significant association was observed between WNV IgM seropositivity and the use of mosquito repellents (p =0.016). Findings from this study necessitate the need for routine surveillance of WNV. More so, infected patients should be closely monitored in order to detect possible associated sequelae.

**Disclosures:**

All Authors: No reported disclosures

